# Histological analysis of neuronal changes in the olfactory cortex during pregnancy

**DOI:** 10.1016/j.heliyon.2024.e26780

**Published:** 2024-02-22

**Authors:** Ken Ichi Matsuda, Tomoki Takahashi, Sae Morishita, Masaki Tanaka

**Affiliations:** Department of Anatomy and Neurobiology, Graduate School of Medical Science, Kyoto Prefectural University of Medicine, Kyoto, Japan

**Keywords:** Olfactory cortex, Dendritic spine, Pregnancy, Estrogen, Oxytocin

## Abstract

Fluctuations in olfactory sensitivity are widely known to occur during pregnancy and may be responsible for hyperemesis gravidarum. These changes are thought to be caused by structural and functional alterations in neurons in response to marked changes of the hormonal milieu. In this study, we examined changes in neurons in the olfactory cortex during pregnancy and after delivery in rats. Dendritic spine densities were measured in the piriform cortex (PIR) and posterolateral cortical amygdala (COApl), which are involved in olfaction. The results showed increased numbers of dendritic spines in the PIR in mid-pregnancy and in the COApl during early and late pregnancy, but not in the motor area of the cerebral cortex, indicating a correlation with changes in olfactory sensitivity during pregnancy. Immunohistochemical analysis of expression of ovarian hormone receptors in these brain regions revealed a decrease in the number of estrogen receptor α-positive cells during pregnancy in the PIR and during pregnancy and the postpartum period in the COApl. Regarding pregnancy-related peptide hormones, oxytocin receptors were expressed in the PIR and COApl, while prolactin receptors were not found in these regions. Accordingly, oxytocin-containing neurites were distributed in both regions. These results suggest that the balance of these hormonal signals has an effect on olfactory sensitivity in pregnant females.

## Graphical abstract

**Figure:**
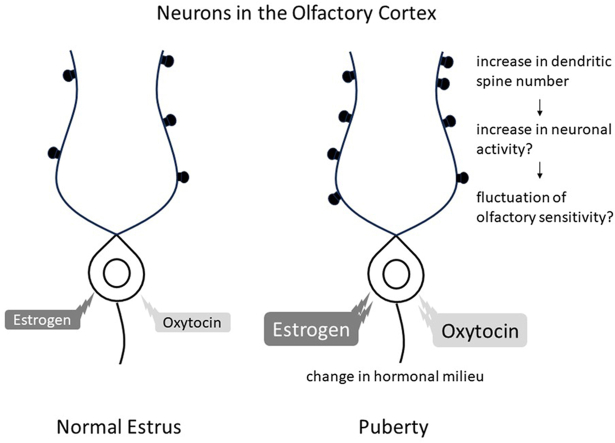

## 1 Introduction

During pregnancy, sensory functions such as smell [1,2], taste [3,4], skin pain [5,6] and itching [7,8] are altered. In particular, alterations in the senses of smell are associated with hyperemesis gravidarum experienced by many women in early and mid-pregnancy [9,10]. In hyperemesis gravidarum, women suddenly develop a strong aversion to smells that did not bother them before pregnancy. The mechanism of this sensory variability has not been clarified, but it may be due to the marked changes in the hormonal milieu associated with pregnancy [11,12]. These endocrine changes may affect the sensitivity of nasal chemoreceptor cells and neuronal activity in the olfactory pathway [1,2]. Accordingly, administration of pregnancy-related hormones such as ovarian steroids [[13], [14], [15], [16]], oxytocin (OT) [17,18] and prolactin (lactogen) [19,20] has been reported to alter olfactory sensitivity significantly in rodent models.

Olfactory information is transmitted from the olfactory epithelium to the olfactory cortex via the olfactory bulb and tract [21,22]. The piriform cortex (PIR) is the largest subregion of the olfactory cortex and consists of three layers [23,24]. Superficial layer I includes axons derived from the olfactory bulb and apical dendrites of neurons, of which cell bodies are localized in layers II and III. Layer II contains the cell bodies of semilunar (SL) cells and superficial pyramidal (SP) cells. SL cells mainly receive axons derived from the olfactory bulb, whereas SP cells receive inputs from intracortical association fibers, as well as axons from the olfactory bulb. Layer III contains cell bodies of deep pyramidal (DP) cells, and distributed basal dendrites of these cells. Layers I and III also contain cell bodies of inhibitory interneurons.

The posterolateral cortical amygdala (COApl) is another olfactory cortex region, but does not have a distinct three-layer structure like the PIR, and has been shown to be involved in transmission of innate olfactory information [25,26]. Sensitivity to innate odor (e.g., predator smell) may change during gestation and postpartum [27], which may be due to fluctuations in the function of neurons in this cortical nucleus.

We previously analyzed neurostructural changes in the central and basolateral nucleus of the amygdala, an emotional control center, during pregnancy and delivery in rats, and found a significant decrease in the number of dendritic spines after birth [28]. In this study, to examine the neurobiological basis of sense of smell during pregnancy and post-delivery, we examined structural changes of neurons in the olfactory cortex (PIR and COApl). Changes in the number of spines were observed using Golgi staining. Then, to verify involvement of pregnancy-related hormone signaling in the neuronal changes, localization of hormone receptors and neurites including the hormones were analyzed immunohistochemically and fluorescently.

## 2 Materials and methods

### 2.1 Animals

The committee for Animal Research of Kyoto Prefectural University of Medicine authorized all animal experimental procedures (Approval number: M2023-164), and the study conformed to international guidelines on the ethical use of animals. Nulliparous female Wistar rats aged 12 weeks and primiparous pregnant Wistar rats were purchased from Shimizu Laboratory Supplies Co (Kyoto, Japan) and housed at 22 °C with a 12-h light/dark cycle in plastic cages with standard bedding and continuous access to food and water. The ovarian cycle of the nulliparous female rats was determined by vaginal smears. At the time of brain sampling, rats were deeply anesthetized by intraperitoneal injection of sodium pentobarbital (100 mg/kg body weight).

### 2.2 Golgi staining and dendritic spine counting

Brains from rats in the estrus phase (Est) in the normal estrus cycle and brains of dams on gestational days 7 (G7), 14 (G14), 21 (G21), and 4 days after delivery (P4) were used as samples. P4 dams were caged with their pups after parturition. Silver impregnation staining was performed using a FD Rapid Golgi Stain Kit (FD NeuroTechnologies, Ellicott City, MD, USA) [28]. The stained coronal sections (150 μm) including anterior PIR (bregma +0.45 to −0.45) or COApl (bregma −2.45 to −3.35) [29] were observed with a microscope (BX50, Olympus, Tokyo, Japan) and images were captured using a CCD camera (DP-21, Olympus). Dendritic spines were counted along the first branch of the apical dendrite of DP, SP and SL neurons in the PIR or the first branch of neurons in the COApl of which the cell body was located in layer II or III. The dendrites can be observed continuously from the cell body to the third branch. Spine density was determined from the number of spines in a 30-μm length. Spines on the apical dendrite (10 μm) of pyramidal neurons in the cerebral cortex (CTX) (M1 and M2 areas) (bregma +0.45 to −0.45) [29] of which the cell body was located in layer III were counted as a control. Thirty neurons from 3 rats (10 neurons/rat) were analyzed for each group and brain area. Spines were classified as mushroom-type, stubby-type and thin-type using previously described criteria [28].

### 2.3 Immunohistochemistry and immunofluorescence

Perfusion, fixation, brain sectioning and immunohistochemical and immunofluorescent staining were performed as described previously [[30], [31], [32], [33]]. Rats (Est, G15, G14, G21 and P4) were transcardially perfused with 0.05 M ice-cold phosphate buffer (PB) (pH 7.5), followed by perfusion fixation with ice-cold 4% paraformaldehyde in 0.05 M PB (pH 7.4). Brains were postfixed with the same fixative at 4 °C overnight and then immersed in 30% sucrose-0.05 M PB at 4 °C for 5–7 days. Coronal sections (40 μm) were stained immunohistochemically using rabbit anti-estrogen receptor α (ERα) antibody (MC20, 1:1000, Santa Cruz Biotechnologies, Dallas, TX) or rabbit anti-progesterone receptor antibody (ab27596, Abcam, Cambridge, UK) at 4 °C overnight. Every other section was collected to avoid double counting of immunoreactive cells. For visualization, a biotin/peroxidase-conjugated streptavidin amplification system (Histofine SAB-PO kit, Nichirei, Tokyo, Japan) and 3,3′-diaminobenzidine tetrahydrochloride (Sigma-Aldrich, St. Louis, MO) were used. Photographs were captured using a CCD camera (DP-21, Olympus) set on the microscope (BX50, Olympus). ERα-immunoreactive (ERα-ir) cells were counted in a rectangular area (0.5 × 1.3 mm) in the PIR and a square area (0.4 × 0.4 mm) in the COApl. Coronal sections (40 μm) were incubated with rabbit anti-OT (20068, 1:2000, ImmunoStar, Hudson, WI) or mouse anti-OT-neurophysin (NP) (1: 1000) [34] at 4 °C overnight, followed by Alexa fluor 488 secondary antibody. Fluorescent images were captured using a BZ-X710 fluorescent microscope (Keyence, Osaka, Japan)

### 2.4 RT-PCR

Brains of Est rats were removed immediately after decapitation. Two brain slices including the PIR (bregma +0.45 to −0.45) or COApl (bregma −2.45 to −3.35) [29] were cut at a thickness of 450 μm using a microslicer (Dosaka EM, Kyoto, Japan), and then tissue fragments were bilaterally isolated using a stainless steel pipe (inner diameter, 0.65 mm) [35,36]. Tissue fragments of the mediobasal hypothalamus (MBH) were collected as an area for use as a positive control. Total RNA was extracted using a RNeasy Micro Kit (Qiagen, Hilden, Germany) and reverse transcribed using ReverTra Ace (Toyobo, Osaka, Japan). PCR (95 °C 30 s; 55 °C 30 s; 72 °C 30 s) was performed using a thermal cycler (Veriti, Applied Biosystems, Foster City, CA) in a 20-μl reaction volume.

For amplification of OT receptor (OT-R) and prolactin receptor (PRL-R) genes, the reaction was conducted over 24 cycles. To avoid non-specific amplification, the reaction products (1 μl) were further amplified for 24 cycles using nested reverse primers. For amplification of glyceraldehyde-3-phosphate dehydrogenase (GAPDH), the reaction was conducted over 40 cycles. The sequences of the primers were: OT-R forward cctgtgttctagaccatcct, reverse ctttcacatagactggcagc, reverse nested agcagcaccctagtaggttt; PRL-R forward aaacattcacctgctggtgg, reverse tctaatgtcaggttccgagg, reverse nested gctctggctcaacgatgtaa; GAPDH forward gccatcactgccactcagaa, reverse gttgctgttgaagtcacagg The final *PCR* products were analyzed by 2% *agarose gel electrophoresis stained by Midori Green (Nippon Genetics, Tokyo, Japan)*.

### 2.5 Statistical analysis

All data are shown as the mean ± SEM. The significance of differences in spine numbers was analyzed by one-way ANOVA followed by a Tukey-Kramer *post hoc* test.

## 3 Results

### 3.1 Dendritic spine density

**Fig. 1:**
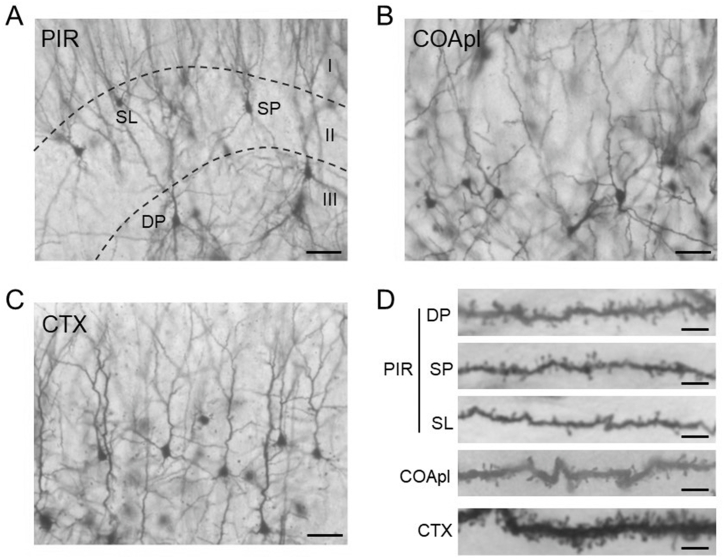
Photomicrographs of representative Golgi-stained brain slices of the piriform cortex (PIR) (A), the posterolateral cortical amygdala (COApl) (B) and the motor area of the cerebral cortex (CTX) (C) in estrus phase rats. (A) Borders of layers I, II and III are indicated by dashed lines. (D) Higher magnification of the first branch of the dendrite. DP: deep pyramidal cell, SP: superficial pyramidal cell, SL: semilunar cell. Scale bars: 100 μm (A); 50 μm (B); 50 μm (C); 5 μm (D).

**Fig. 2:**
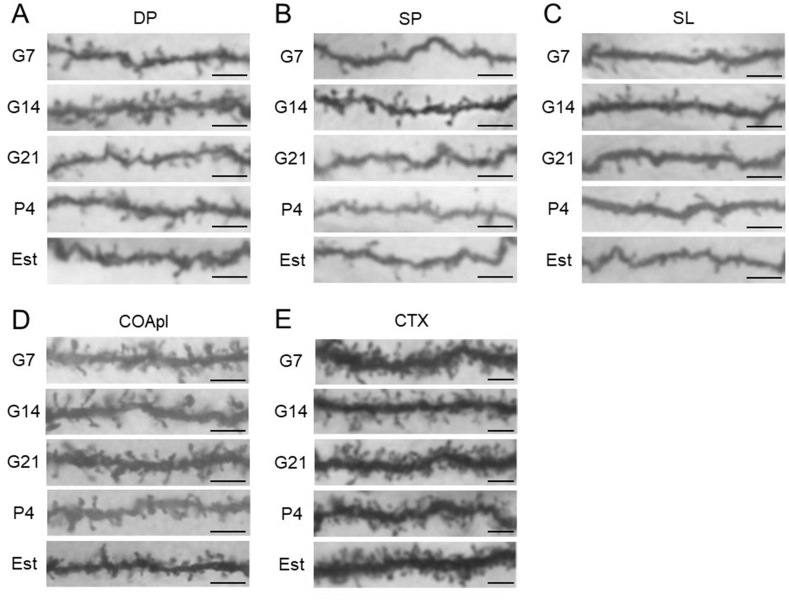
Photomicrographs of representative Golgi-stained dendrites in pregnant [gestational day 7 (G7), gestational day 14 (G14) and gestational day 21 (G21)], postpartum [4 days after delivery (P4)] and normal estrus (Est) rats. (A) Deep pyramidal (DP), (B) superficial pyramidal (SP) and (C) semilunar (SL) cells of the piriform cortex. (D) Posterolateral cortical amygdala (COApl). (E) Motor area of the cerebral cortex (CTX). Scale bars: 5 μm.

**Fig. 3:**
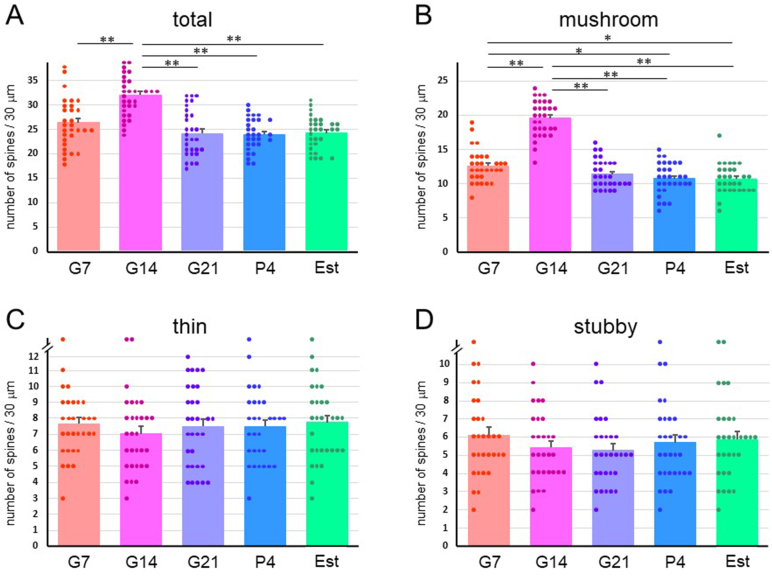
Spine density in a 30-μm dendrite length in deep pyramidal (DP) cells of the piriform cortex in pregnant [gestational day 7 (G7), gestational day 14 (G14) and gestational day 21 (G21)], postpartum [4 days after delivery (P4)] and normal estrus (Est) rats. Densities (mean ± SEM) of (A) total dendritic spines, and (B) mushroom-type, (C) thin-type, and (D) stubby-type spines. **p < 0.01; *p < 0.05.

**Fig. 4:**
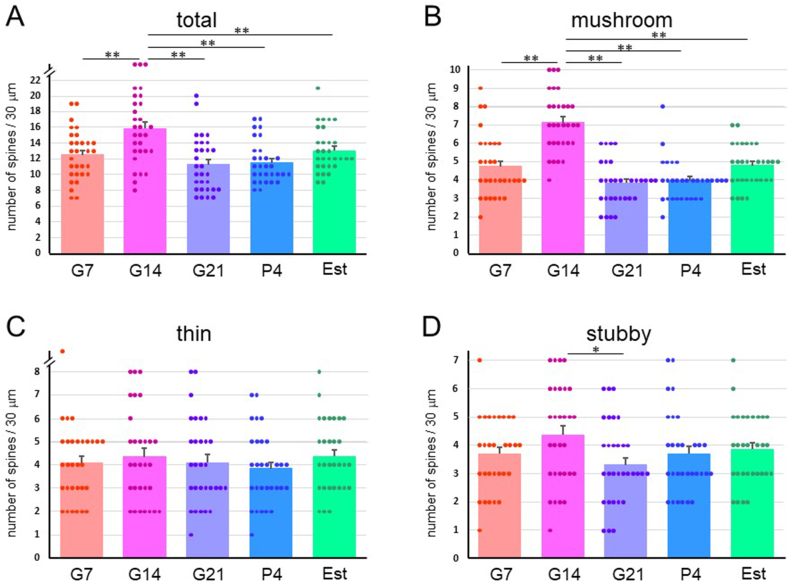
Spine density in a 30-μm dendrite length in superficial pyramidal (SP) cells of the piriform cortex in pregnant [gestational day 7 (G7), gestational day 14 (G14) and gestational day 21 (G21)], postpartum [4 days after delivery (P4)] and normal estrus (Est) rats. Densities (mean ± SEM) of (A) total dendritic spines, and (B) mushroom-type, (C) thin-type, and (D) stubby-type spines. **p < 0.01; *p < 0.05.

**Fig. 5:**
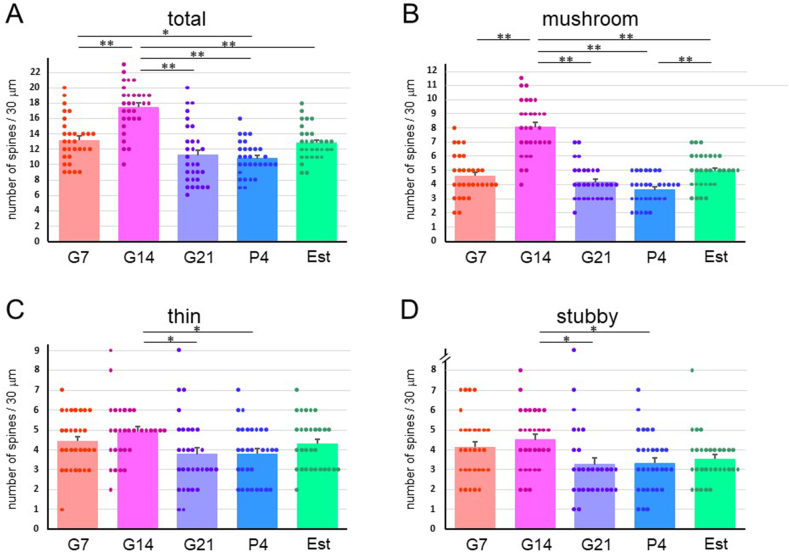
Spine density in a 30-μm dendrite length in semilunar (SL) cells of the piriform cortex in pregnant [gestational day 7 (G7), gestational day 14 (G14) and gestational day 21 (G21)], postpartum [4 days after delivery (P4)] and normal estrus (Est) rats. Densities (mean ± SEM) of (A) total dendritic spines, and (B) mushroom-type, (C) thin-type, and (D) stubby-type spines. **p < 0.01; *p < 0.05.

DP, SP and SL cells in the PIR (Fig. 1A and D) were selected. Images of the first branch of the apical dendrite of G7, G14, G21, P4 and Est rats were captured (Fig. 2A–C) and spine numbers were counted. The total density for all three types of spines (Fig. 3A) and the density of mushroom-type spines (Fig. 3B) in DP cells at G14 were significantly higher than those at G7, G21, P4 and Est. There were no significant differences over time for the thin- and stubby-type spine densities in DP cells (Fig. 3C and D). Similarly, the total and mushroom-type spine densities in SP cells (Fig. 4A and B) and SL cells (Fig. 5A and B) at G14 were significantly higher than those at G7, G21, P4 and Est. Stubby-type spine density in SP cells at G14 was significantly higher than that at G21 (Fig. 4C and D); and thin- and stubby-type spine densities in SL cells at G14 were significantly higher than those at G21 and P4 (Fig. 5C and D).

**Fig. 6:**
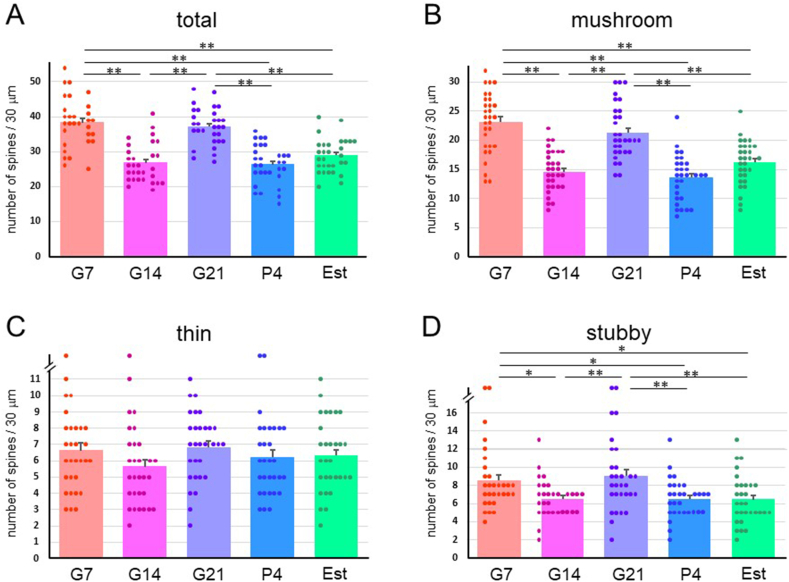
Spine density in a 30-μm dendrite length in neurons of the posterolateral cortical amygdala (COApl) in pregnant [gestational day 7 (G7), gestational day 14 (G14) and gestational day 21 (G21)], postpartum [4 days after delivery (P4)] and normal estrus (Est) rats. Densities (mean ± SEM) of (A) total dendritic spines, and (B) mushroom-type, (C) thin-type, and (D) stubby-type spines. **p < 0.01; *p < 0.05.

**Fig. 7:**
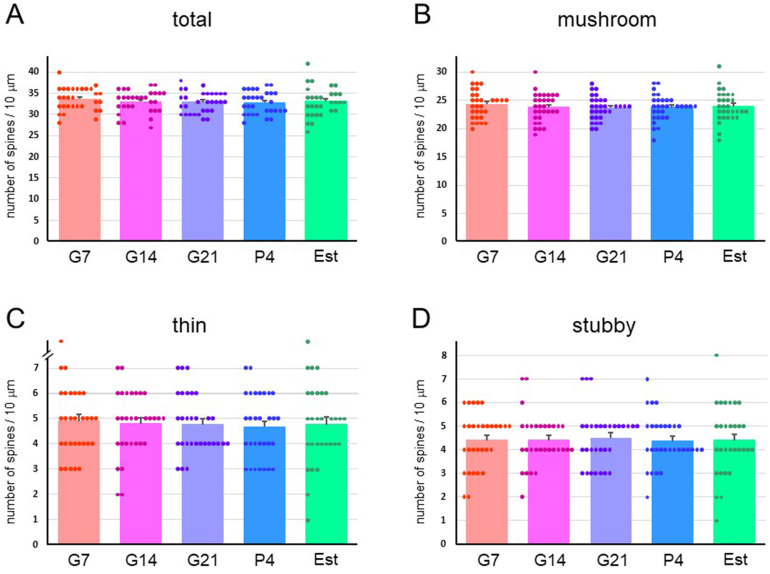
Spine density in a 10-μm dendritic length in the cerebral cortex (CTX) cells in pregnant [gestational day 7 (G7), gestational day 14 (G14) and gestational day 21 (G21)], postpartum [4 days after delivery (P4)] and normal estrus (Est) rats. Densities (mean ± SEM) of (A) total dendritic spines, and (B) mushroom-type, (C) thin-type, and (D) stubby-type spines.

The spine numbers of the first branch of apical dendrites (Fig. 1, Fig. 2D) of neurons in the COApl (Fig. 1B) were also counted. In this analysis, the total, mushroom- and stubby-type spine densities at G7 and G21 were significantly higher than those at G14, P4 and Est (Fig. 6). The spine density of layer III pyramidal cells (Fig. 1, Fig. 2E) in the cerebral CTX M1/2 areas (Fig. 1C) did not differ significantly among the groups (Fig. 7).

### 3.2 Localization of ovarian hormone receptors

**Fig. 8:**
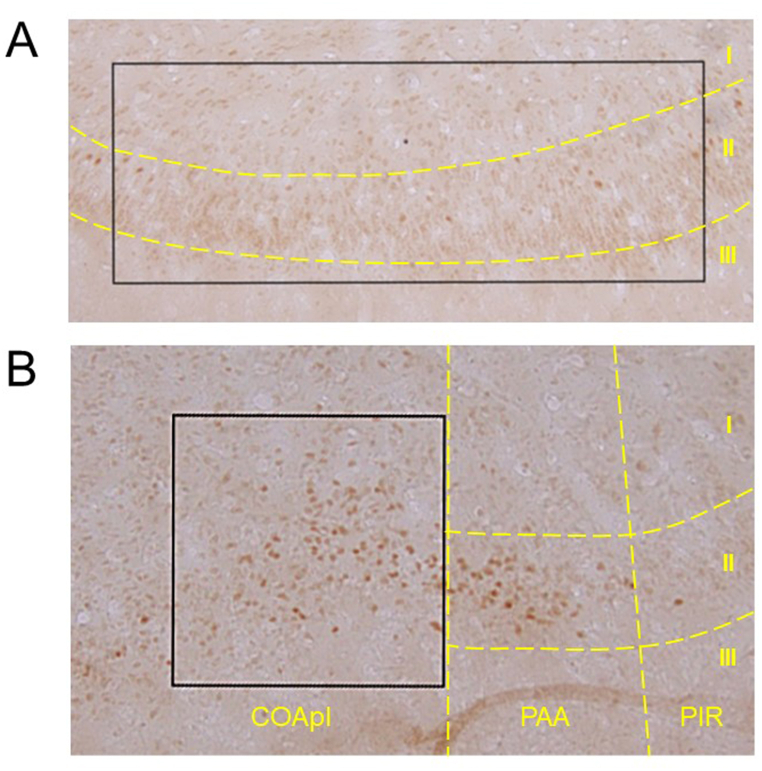
Photomicrographs of immunohistochemical staining for ERα in the piriform cortex (PIR) (A) and posterolateral cortical amygdala (COApl) (B) in estrus phase rats. A rectangular area (0.5 × 1.3 mm) (A) or a square area (0.4 × 0.4 mm) (B) was used for counting nuclear ERα-positive cells. Borders of brain areas and layers I, II and III are indicated by yellow dashed lines. PAA: piriform-amygdaloid area, PIR: piriform cortex. (For interpretation of the references to colour in this figure legend, the reader is referred to the Web version of this article.)

**Fig. 9:**
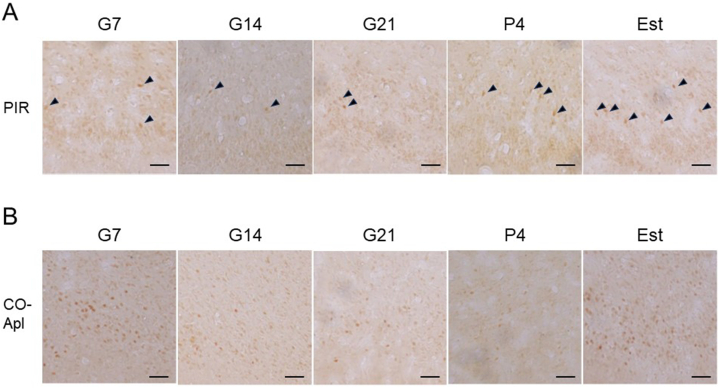
Photomicrographs of immunohistochemical staining for ERα in the piriform cortex (PIR) (A) and posterolateral cortical amygdala (COApl) (B) in pregnant [gestational day 7 (G7), gestational day 14 (G14) and gestational day 21 (G21)], postpartum [4 days after delivery (P4)] and normal estrus (Est) rats. Arrowheads in (A) indicate localization of ERα-immunoreactive cells. Scale bars: 50 μm.

**Fig. 10:**
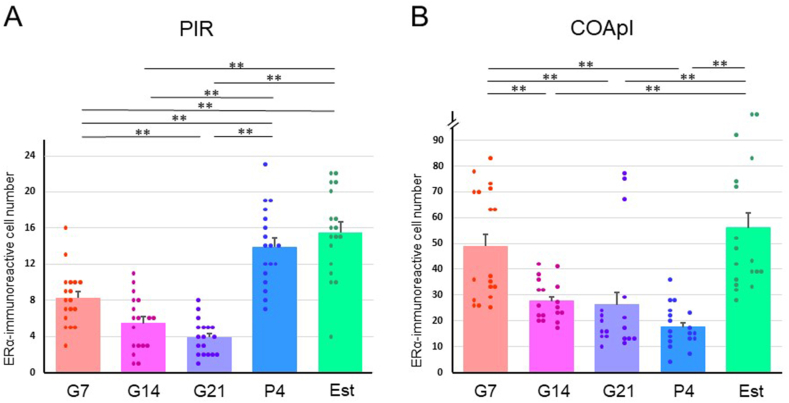
ERα-immunoreactive cell counts in a rectangular area (0.5 × 1.3 mm) in the piriform cortex (PIR) (A) and a square area (0.4 × 0.4 mm) in the posterolateral cortical amygdala (COApl) (B) in pregnant [gestational day 7 (G7), gestational day 14 (G14) and gestational day 21 (G21)], postpartum [4 days after delivery (P4)] and normal estrus (Est) rats. Bar: Mean ± SEM. **p < 0.01.

Localization of ovarian hormone receptors was examined immunohistochemically. ERα-ir was present at low density in most cell bodies in the PIR and COApl (Fig. 8). Cells with high density nuclear ERα-ir were also observed in both olfactory cortices, while nuclear ERα-positive cells were more scattered in the PIR (Fig. 8A) compared to the COApl (Fig. 8B). Distribution of nuclear ERα-ir cells was observed in G7, G14, G21, P4 and Est rats (Fig. 9). The number of nuclear ERα-positive cells was significantly decreased at G7, G14 and G21 compared to P4 and Est in the PIR (Fig. 10A); and at G4, G21 and P4 compared to G7 and Est in the COApl (Fig. 10B). Immunoreactivity of progesterone receptor using a specific antibody was not detected in the PIR or COApl (data not shown).

### 3.3 Expression of receptors for pregnancy-related peptide hormones

**Fig. 11:**
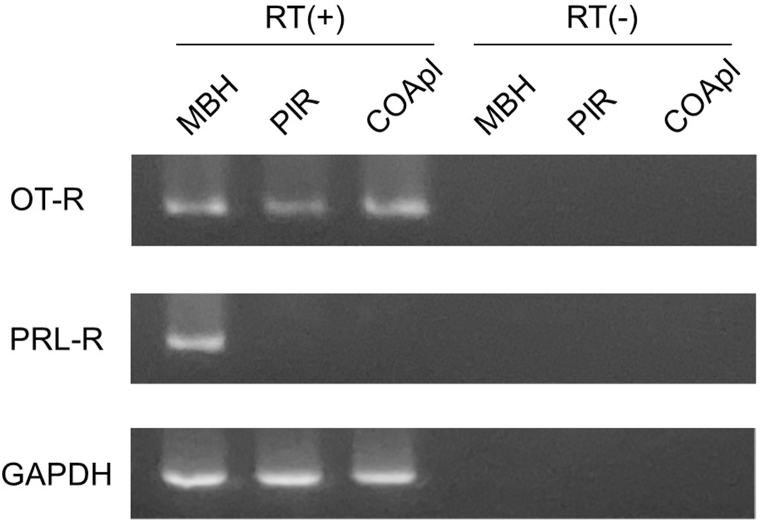
Detection of mRNAs for oxytocin receptor (OT-R), prolactin receptor (PRL-R) and glyceraldehyde-3-phosphate dehydrogenase (GAPDH) in the mediobasal hypothalamus (MBH), piriform cortex (PIR) and posterolateral cortical amygdala (COApl) of normal estrus rats by RT-PCR. PCR products were analyzed by agarose gel electrophoresis. Representative photographs of agarose gel electrophoresis are shown. RT(+): cDNA synthesis in the presence of reverse transcriptase. RT(−): cDNA synthesis in the absence of reverse transcriptase.

Expression of mRNA for receptors for pregnancy-related peptide hormones in the olfactory cortices of Est rats was examined by RT-PCR (Fig. 11). mRNAs for OT-R and GAPDH, a housekeeping gene, were detected in the PIR and COApl, and in the MBH (positive control). In contrast, prolactin (lactogen) receptor mRNA was detected in the MBH, but not in the PIR or COApl.

### 3.4 Localization of OT including neurites

**Fig. 12:**
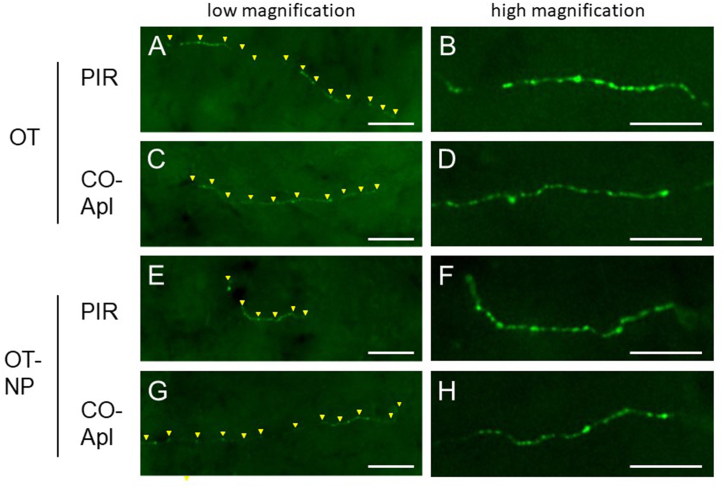
Representative fluorescent images captured using a ×20 (A, C, E, G) or ×40 (B, D, F, H) objective lens for neurites with immunoreactivity for oxytocin (OT) (A–D) and oxytocin-neurophysin (OT-NP) (E–H) in the piriform cortex (PIR) (A, B, E, F) and the posterolateral cortical amygdala (COApl) (C, D, G, H). (A, C, E, G) Yellow arrowheads indicate localization of immunoreactive neurites. Scale bars: 50 μm (G); 20 μm (H). (For interpretation of the references to colour in this figure legend, the reader is referred to the Web version of this article.)

Expression of OT-R in the olfactory cortices indicates that the OT signal is involved in the change of smell sensitivity during pregnancy. Localization of neurites with OT was shown immunofluorescently in the olfactory cortices of Est rats using antibodies specific for OT and OT-NP. A few neurites including OT or OT-NP (0–3 neurites/slice) that had varicosity-like swelling were present in the PIR (Fig. 12A, B, E, F) and COApl (Fig. 12C, D, G, H). The number of fibers positive for OT/OT-NP in each section did not change significantly in the samples during gestation or postpartum (data not shown).

## 4 Discussion

This study shows that dendritic spine density in the olfactory cortex changes during pregnancy in rat. The density did not change in the motor area of the CTX, but did in the PIR and COApl, which may be linked to pregnancy-associated fluctuation of olfactory sensitivity [1,2]. Among the spine types, there was a particular increase in the mushroom-type in both regions. Since the mushroom-type is thought to signify mature spines [37], this suggests activation of neurons during periods of rising spine numbers and subsequent activation of olfactory sensitivity. It is important to examine how this activation of neurons in the olfactory cortex affects downstream neuronal circuits. In the gestation periods when mushroom-type spines increased, thin- and stubby-type spines also tended to increase, indicating that plastic changes in synapse formation occur simultaneously.

In the PIR, DP, SP and SL neurons all had increased numbers of spines in mid-pregnancy. This result may correlate with the occurrence of hyperemesis gravidarum from early to mid-gestation in humans [9,10]. However, humans and rats have different gestation periods and it is also unclear whether rats experience hyperemesis gravidarum during pregnancy. In humans, hormonal changes during pregnancy are more drastic than in rats [11,12]. Because this strong hormonal effect affects the olfactory cortex over a longer gestation period, more marked neuronal changes may occur in humans than in rats, leading to hyperemesis gravidarum.

In the COApl, in contrast, the number of spines increased in early and late pregnancy. Since the PIR and COApl have a reciprocal axonal connection [38], differences in the period of elevated spine number between the olfactory nuclei during gestation may have a positive or negative influence on olfactory sensation. The COApl is known to be involved in the sense of innate odor [25,26]. In rodents, during pregnancy and after delivery, olfactory information becomes increasingly important to detect the smell of predators and competitors of the same or different species and to recognize the scent of pups and colony mates [2]. The finding that the spine number in the COApl is elevated in critical gestational periods may indicate increased maternal sensitivity to protect themselves and their fetus from fatal attacks.

The hypothalamus, the bed nucleus of the stria terminalis and the medial preoptic area are crucial for regulation of maternal behavior [39]. The spinal change in the olfactory cortex during pregnancy shown in this study may be involved in the onset of maternal behavior through the function of these maternal behavior-related brain regions via direct or indirect neural connections.

In pregnancy, the levels of circulating ovarian hormones increase markedly [11,12]. The PIR and COApl have localization of ERα [40], but no expression of progesterone receptors [41], suggesting that estrogen, among the two classes of ovarian hormones, affects olfactory sensitivity through modulation of neural activity. Activation of ERα increases the spine density in hippocampal neurons and improves memory functions [42]. This supports the idea that neurons in the olfactory cortex are temporally activated during pregnancy. In the current study, nuclear ERα-ir was sparsely distributed in the PIR and relatively densely distributed in the COApl. Thus, there may be differences in the response to estrogen in the two regions. Because colocalization of nuclear ERα and glutamic acid decarboxylase 67 immunoreactivities was detected at low frequency (data not shown), most of the nuclear ERα-positive cells in the PIR and COApl were thought to be glutamatergic DP, SP or SL cells and pyramidal cells, respectively. Staining in cell bodies also indicated distribution of membrane ERα, and it is possible that both genomic (via transcriptional regulation) [43] and non-genomic (via a signal transduction cascade) [43,44] actions of estrogen act concertedly on the function of neurons in the olfactory cortex.

The number of nuclear ERα-positive cells was reduced after gestation in both the PIR and COApl, possibly due to negative feedback [43], but with an earlier decrease in the PIR (G7) than in the COApl (G14). Also, the number of ERα-ir cells recovered in the PIR by 4 days postpartum, whereas the reduction persisted in the COApl. Further studies are needed to investigate the underlying mechanism of these differences between the two olfactory cortex regions.

Analysis of expression of receptors for pregnancy-related peptide hormones showed expression of OT-R in both olfactory cortex regions [45] and distribution of OT-positive neurites in these regions. OT may be secreted and exert its effects through a mode called volume transmission, rather than through synaptic transmission [46]. It will be of interest to determine if OT-containing neurites originate from the hypothalamic paraventricular nucleus or suprachiasmatic nucleus.

The oxytocin signal promotes an increase in the number of spines in the hippocampus and, as a result, activates the electrophysiological activity of neurons [47]. Additionally, deletion of oxytocin receptors in the hippocampus inhibits social recognition memory [48]. Since an olfactory cue is important for social recognition [39], oxytocin signaling may be involved in increasing or decreasing the number of spines in the olfactory cortex, thereby affecting sensing of olfactory information. If similar oxytocin-mediated spine changes occur in the hypothalamus, these may also affect hypothalamic endocrine and metabolic axes controlling pregnancy-associated physiology, such as fetal growth and growth and maturation of the mammary glands.

Receptors for prolactin (lactogen), another pregnancy-related peptide, are not expressed in these regions [49]. Although prolactin does not act directly in the PIR and COApl, it may act through synaptic connections via other receptor-positive brain regions. Prolactin stimulates neurogenesis in the subventricular zone during pregnancy in mice [20]. A recent study [50] has shown that in early pregnancy, neurogenesis increases transiently in the subventricular zone, including in subdomains that are usually quiescent. Proliferated cells migrate to a specific region in the olfactory bulb, which is different from that during non-pregnancy, and then differentiate into interneurons by late-pregnancy to be involved in perception of offspring odor after delivery. It is possible that this neurogenesis-associated reorganization of neural circuits in the olfactory bulb is correlated with the increase in the number of synapses in the PIR during mid-pregnancy and/or in the COApl during early- and late-pregnancy, and that prolactin signaling has an influence in the initial step of this reorganization.

The results of this study suggest that a balance of multiple hormonal signals alter neuronal activity via changes in the synapse number in the olfactory cortex and affect olfactory sensing during pregnancy. In addition to the endocrine environment, there are other factors that fluctuate in the maternal body during pregnancy, such as the immune response [51,52] and nutritional status [53,54]. Analyses focusing on the interaction between these factors and endocrine responses are required in a future study. During pregnancy, there are changes in both the sense of smell and other sensory sensitivities [1,3,6,8]. Thus, it is also of interest to examine similarities and differences in the mechanisms of fluctuations of each sense during pregnancy.

## Funding

This work was supported by 10.13039/501100001691Japan Society for the Promotion of Science KAKENHI Grant Number 20K09625.

## Data availability

All data used in the study are described in the article.

## CRediT authorship contribution statement

**Ken Ichi Matsuda:** Writing – original draft, Visualization, Validation, Project administration, Investigation, Funding acquisition, Formal analysis, Data curation, Conceptualization. **Tomoki Takahashi:** Visualization, Validation, Investigation. **Sae Morishita:** Visualization, Validation, Investigation. **Masaki Tanaka:** Validation, Supervision.

## Declaration of competing interest

The authors declare that they have no known competing financial interests or personal relationships that could have appeared to influence the work reported in this paper.
